# Function-Focused Care in the Dutch Nursing Home Setting: Past and Current Practices

**DOI:** 10.1093/geroni/igaa057.2151

**Published:** 2020-12-16

**Authors:** Stan Vluggen, Silke Metzelthin, Janneke de Man-van Ginkel, Getty Huisman de Waal, Sandra Zwakhalen

**Affiliations:** 1 Maastricht University, Maastricht, Limburg, Netherlands; 2 Julius Center for Health Sciences and Primary Care, UMC Utrecht, Utrecht, Utrecht, Netherlands; 3 Radboud University Medical Center, Nijmegen, Gelderland, Netherlands

## Abstract

In recent years, Function Focused Care (FFC) interventions have been developed and implemented in Dutch home care, nursing homes and acute care. These interventions aimed to train nursing staff to adapt their level of support to the capabilities of elderly and to maintain/optimize their self-reliance and physical functioning. After synthesizing knowledge and experiences from the existing FFC-interventions, an advanced FFC-intervention was developed for application in long-term care: ‘SELF’. SELF comprises seven interactive sessions, is theoretically grounded, primarily focuses on behavior change in nursing staff, is tailored to the ward’s needs, is multidisciplinary in nature, and focuses on all care interactions. Currently, SELF is pilot-tested in one psycho-geriatric ward with 17 nursing staff members. Questionnaires on outcome-expectations, self-efficacy and intention to stimulate elderly are assessed at baseline and after 3 months. Field notes and interviews are used to assess its feasibility and acceptability. Experiences will guide the further refinement of ‘SELF’. Part of a symposium sponsored by Nursing Care of Older Adults Interest Group.

